# A conversation with Loren Miller, MD, MPH, professor and chief, Division of Infectious Diseases, Lundquist Institute at Harbor-UCLA

**DOI:** 10.1017/cts.2025.10045

**Published:** 2025-05-30

**Authors:** Clinical Research Forum

**Affiliations:** Clinical Research Forum, Washington DC, USA

## Top 10 clinical research achievement awards Q & A

This article is part of a series of interviews with recipients of Clinical Research Forum’s Top 10 Clinical Research Achievement Awards. This interview is with Loren Miller, MD, MPH, Professor of Medicine at the David Geffen School of Medicine at University of California, Los Angeles, and Chief in the Division of Adult Infectious Diseases at Harbor-UCLA Medical Center. Dr Miller’s research focuses on the epidemiology, prevention, and treatment of common infectious diseases, as well as health services research approaches to studying delivery of care to patients with suspected or documented infectious diseases. He received a 2024 Top 10 Clinical Research Achievement Award for “Protect Trial: Protecting Nursing Home Residents from Infection and Hospitalization.” *The interview has been edited for length and clarity.*

## When did you first become interested in clinical research?

After medical school, I did a fellowship in infectious diseases and health services research, which is the study of the health care delivery system. Back then, I was interested in becoming a clinician educator and had no intention of going into research, but over the course of the health services fellowship, I got involved with a project investigating how people with HIV and AIDS complied with their treatment regimens. I really enjoyed being part of that team. I knew that what I was doing was important, my mentors kept encouraging me, and the next thing I knew, I was writing grants – and getting grants to do more research. I wound up still being able to do education, but now I spend my time primarily on research.

## What led to your focus on community-acquired infections?

There was an outbreak of community-acquired methicillin-resistant Staphylococcus aureus (MRSA) infections in our area, and at the time, there were all sorts of unanswered questions about how these infections spread and could be managed. Again, I was fortunate to have colleagues who encouraged me to apply for grants to research this, and that started a career shift toward staph infections and infection prevention overall. I want to make the point that infection prevention is the priority. By the time someone needs treatment for an infection, they’re already suffering. What we need to do is prevent, so we don’t have to treat. That’s what led us to do this project on infection prevention and nursing homes, focusing on MRSA and other antibiotic-resistant and difficult-to-treat infections.

## What did the award-winning trial show?

Our trial found that in nursing homes, switching routine bathing soap with chlorhexidine antiseptic wash plus the twice-daily administration of nasal iodophor for five consecutive days every other week significantly reduced the risk of transfer to a hospital due to infection.

## Why is this result important?

Our findings suggest that the prevention of serious infection in nursing homes can be facilitated with a relatively simple intervention. That’s important because nursing home residents are at elevated risk for infection, and those infections can result in hospitalization, especially if they involve multidrug-resistant organisms. Plus, antibiotic-resistant infections are increasingly difficult to treat, with this horrible cycle of escalating the antibiotics we use and then the pathogens escalating their resistance. If we could have fewer infections and use less antibiotics in general, then the risk of developing drug-resistant infections would decrease. As I mentioned, an ounce of prevention is worth a pound of cure, and given the large and increasing numbers of persons cared for in nursing homes, this intervention could prevent a substantial amount of hospitalization-associated morbidity and save health care resources.

## What is the next step for this research?

The next step is to implement this intervention on a wider scale. There were 24 nursing homes in our study, and now we’d like to roll this out to more, across larger regions. To do that, we need to partner not only with the facilities, but with public health organizations, medical societies, and so forth. We need to identify both the facilitators and challenges to implementation.

## What could be potential challenges?

Cost could be a barrier – not because of chlorhexidine or nasal iodophor (they’re both relatively inexpensive and available over the counter), but because there could be increased costs associated with the administration of nasal povidone. Also, using chlorhexidine for bathing can be challenging because it reacts with some commonly used lotions. That means residents need to be checked to ensure they’re only using lotions that don’t interact. Another issue is that residual chlorhexidine on bed sheets can result in staining if bleach is used in the laundry. So, nursing homes using this intervention have to make sure their laundry vendors are using alternatives to bleach. Challenges like these are not overly complicated, but they may require training and support for nursing home staff.

## What advice would you give someone starting their career in clinical research?

First, I would tell them that of all the people I know who are in research, no two have followed the same path. In fact, everyone’s path is so radically different that there’s no need to worry that there is a “best” path. Second, I would say to follow your heart and pursue research topics that you’re truly interested in. You want to be able to wake up in the morning and be excited about going to work. My other piece of advice is to talk to people. I’ve learned a lot by reading books and journal articles, but I’ve gotten the most important information from just talking. It’s surprising how often even hallway conversations can lead to incredible insights, great ideas, and new connections. There’s nothing more invaluable than interacting with your colleagues. You learn so much from those conversations, even if you’re doing research in vastly different fields.

## It sounds like you enjoy practicing team science

Absolutely. Although to me, the term “team science” seems like a redundancy. Science is a team sport. No one can do it alone. No one group can do it alone. You need to involve people from different backgrounds. They bring ideas and new perspectives to make your project more meaningful – and that includes community partners, as well.

## What role do community partners play?

Community partners, such as patient representatives, disease advocates, etc., offer unique insights, ones you wouldn’t get from your scientific colleagues. Understanding those different perspectives helps the research be more applicable. For example, earlier today, I was discussing a community partner for an upcoming project about infection prevention and dialysis, and it became clear that we actually need more than one community partner. We knew we wanted to learn about dialysis from the patient perspective. But a lot of dialysis patients have caregivers, so we realized we need to include the caregiver perspective, as well. So now our team is expanding. We want to be sure to include as many relevant perspectives as we can.

## What hobbies do you enjoy and how do they impact your work?

I enjoy being outside and taking walks with my wife, my family, and our dog. I am also an avid long-distance unicyclist, and I especially like to unicycle up hills. There’s only one gear, so it takes a lot of effort, and I find that’s good to silence the rest of the world. It takes me away from research for a while and clears my thoughts. I rode a unicycle once when I was a kid, and I remember liking it. But I didn’t really learn to ride until I was 43. Now I’m part of a club and even do off-road or “Muni,” which is short for mountain unicycling.

